# Glucose control and mortality from lower respiratory tract infections in patients with diabetes: evidence from real-world data

**DOI:** 10.1186/s12879-026-13045-8

**Published:** 2026-03-14

**Authors:** Youngmin Shin, Sang Jun Eun, Ju-Mi Lee

**Affiliations:** 1 The Graduate School of Public Health, Chungnam National University, Daejeon, Korea; 2 Department of Preventive Medicine, Chungnam National University College of Medicine, 266 Munhwa-ro, Jung-gu, Daejeon, 35015 Korea

**Keywords:** Diabetes mellitus, Blood glucose, Respiratory tract infections, Mortality

## Abstract

**Background:**

Diabetes mellitus increases both susceptibility to and severity of lower respiratory tract infections (LRTIs). However, the quantitative relationship between the range of glucose control and mortality from LRTIs in patients with diabetes has not been well defined. This study evaluated the association between fasting blood glucose (FBG) at the health check-up closest to the LRTI diagnosis and subsequent LRTI mortality in patients with diabetes, using data from the Korean National Health Insurance Service–National Sample Cohort (NHIS-NSC).

**Methods:**

This study included 4,514 adults with diabetes diagnosed with pneumonia (J12–J18) or influenza (J09–J11) in the cohort between 2002 and 2019. Mortality after LRTI diagnosis was identified using claims and cause-of-death records. FBG from the health check-up closest to the LRTI diagnosis was classified into three categories (< 80, 80–130, ≥ 130 mg/dL) based on Korean Diabetes Association recommendations. Logistic regression was performed to estimate the association between FBG categories and mortality after adjustment for age, sex, smoking status, physical activity, body mass index, hypertension, income, cardiovascular disease, and duration of diabetes. Dose–response relationships in group3(FBG ≥ 130 mg/dl) were evaluated using linear and restricted cubic spline models.

**Results:**

Among the study population, 212 patients (4.7%) died from LRTIs. Compared with those with target-range FBG (80–130 mg/dL), patients with FBG ≥ 130 mg/dL had a significantly higher risk of mortality (adjusted odds ratio [aOR] 1.54; 95% confidence interval [CI] 1.11–2.14). This association remained robust after excluding outliers (FBG > 383 mg/dL) and in extended adjustment models. A clear dose–response relationship was observed between FBG and mortality risk. Relative to target-range FBG, mortality risk increased 1.4-fold at 160–189 mg/dL and rose sharply to 2.5-fold at ≥ 190 mg/dL.

**Conclusions:**

Real-world data showed that elevated FBG in patients with diabetes increased the risk of mortality from LRTI. This study not only demonstrates, using real-world data, the importance of adequate glycemic control in patients with diabetes, but also identifies a threshold at which mortality risk rises sharply. These findings emphasize that appropriate glycemic control may reduce infection-related mortality and should be considered a critical component of public health strategies for diabetic populations.

**Supplementary Information:**

The online version contains supplementary material available at 10.1186/s12879-026-13045-8.

## Background

Diabetes mellitus (DM) is strongly associated with an increased risk of various infectious diseases, mainly due to chronic hyperglycemia that impairs host immune defense. Elevated glucose levels can suppress neutrophil phagocytic activity and cytokine production and impair the function of CD4⁺ T cells and natural killer cells, thereby weakening both innate and adaptive immunity [1–3]. These immunological mechanisms provide a biological explanation for the greater susceptibility of individuals with diabetes to infections.

A growing body of epidemiological evidence has shown that poor glycemic control in diabetic patients increases both the incidence and mortality of lower respiratory tract infections (LRTIs), including pneumonia and influenza. In diabetic individuals, aspiration pneumonia can occur more frequently because oral bacteria are more likely to reach the lungs, and impaired mucociliary clearance contributes to higher vulnerability to respiratory infections [4–6]. During the H1N1 pandemic, diabetic patients had a 4.29-fold higher risk of intensive care unit admission [7], and diabetes was also linked to increased severity and mortality during coronavirus disease 2019 (COVID-19) outbreaks [8]. Similarly, older adults (≥ 65 years) with diabetes experienced higher rates of hospitalization, intensive care, and death from influenza [9]. A population-based cohort study from Denmark reported 30-day and 90-day mortality ratios of 1.16 and 1.10, respectively, following pneumonia admission [10]. Moreover, elevated hemoglobin A1C (HbA1c) levels have been significantly correlated with a higher risk of infection, underscoring the role of glycemic control as a key determinant of infection outcomes [11].

In South Korea, the prevalence of diabetes has been increasing rapidly. As of 2022, approximately 14.8% of adults aged ≥ 30 years and 28.0% of older adults aged ≥ 65 years have diabetes, while 41.1% and 47.7% are classified as having prediabetes, respectively [12, 13]. These figures indicate that diabetes represents a major chronic disease requiring targeted public health interventions.

In addition, the resurgence of respiratory infections [14, 15] after the COVID-19 pandemic (2020–2023) [16] highlights the renewed importance of understanding infection burden in vulnerable populations such as those with diabetes.

Therefore, this study aimed to quantify the association between glycemic control and mortality from LRTIs—including pneumonia (J12–J18) and influenza (J09–J11)—in patients with diabetes, using data from the Korean National Health Insurance Service–National Sample Cohort (NHIS-NSC). We hypothesized that inadequate glycemic control increases infection-related severity and conducted this analysis to test that hypothesis. Through this investigation, we highlight the public health relevance of effective glucose management in improving infection outcomes among individuals with diabetes.

## Methods

### Data source and study population

The NHIS-NSC is a nationwide real-world cohort derived from Korea’s single-payer National Health Insurance (NHI) system, which covers the entire Korean population. It includes comprehensive information on demographics, diagnoses, prescriptions, hospital admissions and discharges, national health screening results, and mortality records. Approximately one million individuals (about 2% of the total population) were selected through a probability sampling method to form a nationally representative sample, which is continuously followed over time based on changes in insurance eligibility and healthcare claims data [17]. 

Lifestyle-related variables, including smoking status, alcohol consumption, and physical activity, were obtained from the standardized questionnaires administered as part of the National Health Screening Program conducted by the NHIS of Korea. The structure and data collection procedures of these health screening questionnaires have been described in detail in previous publications, and relevant items were utilized for the present analysis [17, 18]. 

This study used data from the NHIS-NSC collected between 2002 and 2019 (Research approval No. NHIS-2025-06-2-058). Among 1,137,861 adults aged ≥ 20 years with available annual health examination records during the study period, individuals were initially screened for diabetes based on either [1] a previous physician diagnosis of diabetes or current use of antidiabetic medication, or [2] FBG ≥ 126 mg/dL at any health screening. A total of 102,913 individuals met at least one of these criteria.

From this diabetes-defined cohort, we identified 4,514 individuals whose claims records indicated a primary diagnosis of LRTI—specifically pneumonia (ICD-10 J12–J18) or influenza (J09–J11)—occurring after the onset of diabetes. These individuals constituted the final analytic population.

### Study variables and definitions

#### Duration of diabetes and FBG as an indicator of glycemic control

FBG levels, used as an indicator of glycemic control, were obtained from the most recent National Health Screening examination conducted before the month of LRTI diagnosis. Because health screening data in the NHIS-NSC are available only at the year–month level, this approach was used to ensure that FBG measurements reflected glycemic status preceding the infection episode. The duration of diabetes was calculated as the time from the first verified diabetes diagnosis to the date of infection (within 30 days). FBG levels were categorized into three groups according to glycemic targets recommended by the Korean Diabetes Association [12, 19, 20]:

- Group 1: <80 mg/dL (below target).
- Group 2: 80–130 mg/dL (within target, well controlled group).
- Group 3: ≥130 mg/dL (above target, poorly controlled group).

#### Respiratory infection and episode definition

Respiratory infections were identified using ICD-10 codes for pneumonia (J12–J18) and influenza (J09–J11). Only infections that occurred after the earliest confirmed evidence of diabetes -either a prior physician diagnosis or diabetes detected in health screening- were included in the analysis.

An infection episode was defined based on the date of the first diagnosis, and repeated diagnoses with the same ICD-10 code within 90 days were considered part of the same episode [21]. After the conclusion of an episode (based on the last claim date), an additional 90-day window was applied, during which any repeated diagnosis was also regarded as the same episode. This approach was used to avoid multiple counting of repeated or overlapping diagnoses in the same individual.

When multiple episodes occurred in one patient, only the episode with the highest severity was retained to ensure a one-person–one-episode analytic structure and maintain statistical independence.

#### Study outcome

Clinical severity was categorized as death, intensive care unit (ICU) admission, general hospitalization, or outpatient care. Hospitalizations were further classified as long-term (≥ 7 days) or short-term (< 7 days) [20, 22–24]. The primary outcome of this study was mortality attributable to pneumonia or influenza, defined as: (1) death occurring during healthcare encounters related to the infection, and (2) death not occurring during the healthcare encounter but recorded within 30 days after the end of the infection-related healthcare service (hospital discharge or completion of outpatient care), with pneumonia or influenza listed as the cause of death in the Korean Standard Classification of Diseases (KCD) mortality records in the NHIS-NIC.

#### Demographic and lifestyle variables

Demographic characteristics included age, sex, and household income level. Age was categorized into eight groups: 20–29, 30–39, 40–49, 50–59, 60–69, 70–79, 80–89, and ≥ 90 years. Income level was divided into ten deciles, from the lowest (1st decile) to the highest (10th decile) [25]. Lifestyle behaviors included smoking status (non-smoker, former smoker, current smoker) and exercise frequency (none, 1–2 times/week, 3–4 times/week, 5–6 times/week, almost daily).

#### Anthropometric and clinical variables

Body mass index (BMI) was calculated from health examination data and classified as underweight (< 18.5 kg/m²), normal (18.5–22.9 kg/m²), overweight (23.0–24.9 kg/m²), and obese (≥ 25.0 kg/m²) [26]. Hypertension was defined as systolic blood pressure ≥ 140 mmHg, diastolic blood pressure ≥ 90 mmHg, or a recorded diagnosis/use of antihypertensive medication [27].

#### Other comorbidities

The presence of cancer, pulmonary tuberculosis, chronic kidney disease, chronic obstructive pulmonary disease and cardiovascular disease was identified using self-reported medical history information from the NHIS-NSC health examination database.

However, only 5 individuals (0.11%) reported a history of pulmonary tuberculosis, and 84 individuals (1.86%) reported a history of cancer or other chronic diseases, including those currently undergoing treatment. (There were 108 cases of CKD (2.39%) and 272 cases of COPD (6.03%).) Because these conditions were extremely rare and could lead to statistical instability, individuals with these comorbidities were excluded from the main analysis. Nevertheless, these variables were included in the sensitivity analysis to evaluate the robustness of the adjusted models.

### Statistical analysis

All statistical analyses were performed using SAS Enterprise Guide version 8.3 (SAS Institute Inc., Cary, NC, USA). Because the NHIS-NSC data are based on a simple random sample rather than a complex design, no sample weighting was applied.

#### Basic analysis

Differences in sociodemographic and health behavior characteristics across FBG groups were assessed using the chi-square test for categorical variables and the t-test or one-way ANOVA for continuous variables.

Mortality associated with LRTI was evaluated using logistic regression. Odds ratios (ORs), adjusted odds ratios (aORs), and 95% confidence intervals (CIs) were calculated. Confounders- including age, sex, smoking status, physical activity, BMI, hypertension, household income (decile), duration of diabetes (log-transformed, months), and cardiovascular disease- were simultaneously included in a fully adjusted multivariable model.

To evaluate dose–response patterns among individuals with elevated FBG (≥ 130 mg/dL, poorly controlled group), this category was further divided into 130–159 mg/dL, 160–189 mg/dL, and ≥ 190 mg/dL. FBG was also analyzed as a continuous variable using both linear models and restricted cubic spline (RCS) models to identify potential non-linear associations with mortality.

#### Sensitivity analysis

Three types of sensitivity analyses were conducted.

##### Outlier exclusion

Values exceeding Q3 + 1.5×IQR were removed, and the results before and after exclusion were compared.

##### Alternative comorbidity adjustment

Because the prevalence of these comorbidities was extremely low—cancer 1.86% (84 cases), pulmonary tuberculosis 0.11% (5 cases), chronic kidney disease/renal failure 2.39% (108 cases), and COPD diagnosed within the past 3 years 6.03% (272 cases)—they were excluded from the main model to avoid statistical instability. However, they were included in additional sensitivity analyses to evaluate robustness and consistency. In all analyses, a two-sided p-value < 0.05 was considered statistically significant.

### Ethics approval and consent to participate

This study was reviewed by the Institutional Review Board (IRB) of Chungnam National University (approval No. 202505-SB-084-01) and was exempted from full ethical review. The requirement for informed consent was waived because the dataset used (NHIS-NSC) consists of fully de-identified secondary data provided by the NHIS, ensuring that no individual participant can be identified. The study was conducted in accordance with the principles of the Declaration of Helsinki. All analyses were performed using publicly available data released for research and public health purposes.

## Result

### Baseline characteristics

Among the 4,514 diabetic patients diagnosed with LRTI, the distribution of demographic and health-related characteristics differed significantly across FBG categories (Table 1).

Income level, pulmonary tuberculosis, CKD, COPD, cancer and other disease are presented in Supplementary Table 3. CKD, cancer and other disease showed significant differences across the groups (*p* < 0.05).

**Table 1:** Baseline characteristics of diabetic patients with respiratory infection, by FBG categories

Variables	Total (*n* = 4514)	Group 1 (*n* = 285)	Group 2 (*n* = 2523)	Group 3 (*n* = 1706)	*p*-value
Age (years), mean ± SD	60.25 ± 13.05	60.23 ± 14.61	60.76 ± 13.30	59.50 ± 12.36	0.0088^*^
Sex, female	1994(44.17)	114(40.00)	1114(44.15)	746(43.73)	0.3044
Smoking status					
Never smoker	2787(63.69)	152(53.33)	1550(61.43)	1085(63.60)	< 0.0001^*^
Former smoker	594(13.58)	46(16.14)	392(15.54)	156(9.14)	
Current smoker	994(22.73)	74(25.96)	501(19.86)	419(24.56)	
Physical activity					
None	2114(48.35)	126(44.21)	1041(41.26)	947(55.51)	< 0.0001^*^
1–2 times/week	734(16.79)	39(13.68)	384(15.22)	311(18.23)	
3–4 times/week	475(10.87)	36(12.63)	304(12.05)	135(7.91)	
5–6 times/week	256(5.86)	18(6.32)	184(7.29)	54(3.17)	
Almost daily	793(18.14)	54(18.95)	528(20.93)	211(12.37)	
Hypertension					
Yes	813(18.01)	47(16.49)	550(21.80)	216(12.66)	< 0.0001^*^
No	3701(81.99)	238(83.51)	1973(78.20)	1490(87.34)	
BMI (kg/m^2^), mean ± SD	24.51 ± 3.40	24.03 ± 3.49	24.55 ± 3.36	25.54 ± 3.43	0.0452^*^
Cardiovascular disease					
Yes	948(21.00)	36(12.63)	434(17.20)	478(28.02)	< 0.0001^*^
No	3566(79.00)	249(87.37)	2089(82.80)	1228(71.98)	

Note. Data were expressed as mean ± SD or number (%). FBG was classified into three categories (< 80, 80–130, and ≥ 130 mg/dL) according to Korean Diabetes Association recommendations

### Severity and hospital stay of lower respiratory tract infections according to fasting blood glucose category

The severity distribution of LRTIs differed significantly across FBG categories (*p* = 0.0076). Higher FBG levels were associated with a progressively greater number and proportion of deaths, with the highest mortality observed in Group 3 (≥ 130 mg/dL). Among all 4,514 patients, 212 deaths occurred (overall mortality 4.70%). Specifically, mortality rates were 2.46% in Group 1 (< 80 mg/dL; 7 of 285), 3.84% in Group 2 (80–130 mg/dL; 97 of 2,523), and 6.33% in Group 3 (≥ 130 mg/dL; 108 of 1,706).

In contrast, the mean length of hospital stay among patients requiring admission did not differ significantly across FBG groups (*p* = 0.1699). The mean duration of hospitalization was 11.73 ± 8.12 days in Group 1, 10.78 ± 6.12 days in Group 2, and 11.77 ± 8.70 days in Group 3.

### Association between FBG categories and LRTI mortality in logistic regression analysis

Multivariable logistic regression demonstrated a significant association between FBG categories and mortality among patients with diabetes diagnosed with LRTI. Multicollinearity was not detected in any variable, with all variance inflation factor (VIF) values below 5. Model performance was acceptable, with adequate discrimination and calibration based on the AUC, Hosmer–Lemeshow test, and Brier score. The ROC curve further confirmed good discriminatory ability (Supplementary Figure S1). The Nagelkerke R² of 0.24055 indicated a moderate level of explanatory power.

Compared with group 2, group 1 showed no statistically significant difference in mortality risk (aOR 0.526, 95% CI 0.225–1.275; Table 2). In contrast, individuals in group 3 had a significantly higher mortality risk than those in group 2 (aOR 1.542, 95% CI 1.113–2.136).

Absolute mortality rates were 2.24% in Group 1, 3.85% in Group 2, and 6.00% in Group 3, with an absolute risk difference of + 2.15% points for Group 3 compared with the reference category (Supplementary Table S4).

#### Sensitivity analyses

Three sensitivity analyses were performed to evaluate the robustness of findings.

##### Outlier exclusion

After removing 28 outliers (FBG > 383 mg/dL), the increased mortality risk in Group 3 remained consistent (aOR 1.542, 95% CI 1.111–2.140) (Supplementary Table S5).

##### Adjustment for additional chronic conditions

In this sensitivity analysis, individuals with cancer, pulmonary tuberculosis, CKD, or COPD—who were excluded from the main analysis—were included, and these four conditions were additionally adjusted for as confounders. The results showed a similar magnitude of association (aOR 1.551, 95% CI 1.118–2.151) (Supplementary Table S6).

##### Combined outlier exclusion + additional comorbidities

Results remained stable, with an aOR of 1.556 (95% CI 1.120–2.163) for Group 3 (Supplementary Table S7).

All sensitivity analyses consistently confirmed the robustness of the association between higher fasting blood glucose levels and increased mortality (Supplementary Tables S5–S7).

**Table 2:** Association between FBG categories and LRTI mortality in diabetes

Group	Number of participants (*n*)	Number of death (*n*, %)	Crude		Adjusted	
			ORs	(95% CI)	ORs	(95% CI)
Group 1	285	7(2.46)	0.630	(0.290–1.370)	0.526	(0.225–1.275)
Group 2	2523	97(3.84)	1.00		1.00	
Group 3	1706	108(6.33)	1.690	(1.276–2.240)	1.542	(1.113–2.136)

Note. Multivariable logistic regression analysis was performed after adjusting for age, sex, smoking status, physical activity, BMI, hypertension, household income decile, duration of diabetes (log-transformed, months), and cardiovascular disease. FBG was classified into three categories (< 80, 80–130, and ≥ 130 mg/dL) according to Korean Diabetes Association recommendations

### Dose–response relationship between FBG and LRTI mortality

**Fig. 1:**
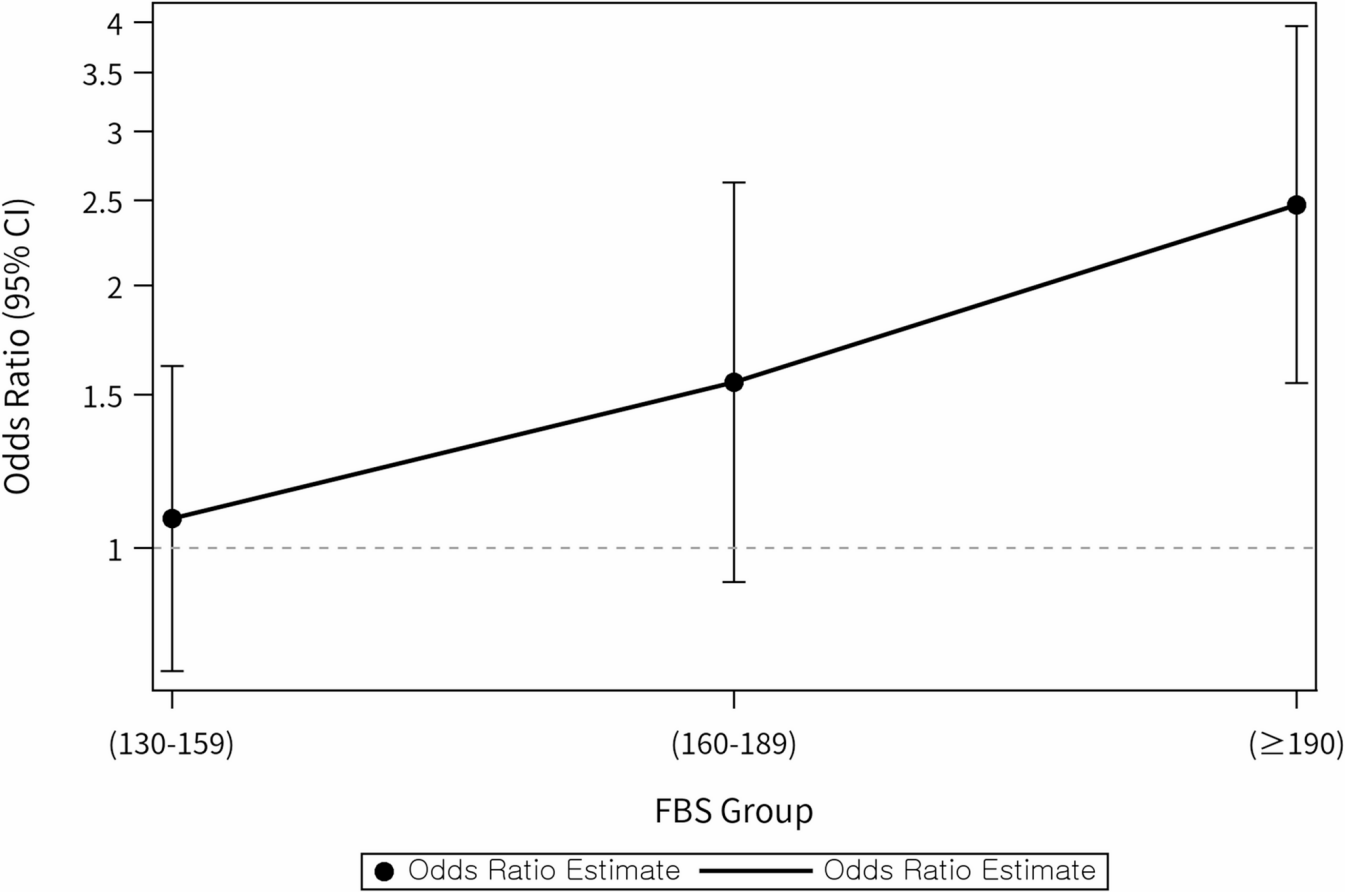
Dose–response relationship between FBG and LRTI mortality in the poorly controlled FBG group. Note. Adjusted odds ratios with 95% confidence intervals derived from multivariable logistic regression across FBG categories. The reference category was 80–130 mg/dL (well controlled group). Covariates included age, sex, smoking status, physical activity, BMI, hypertension, household income decile, duration of diabetes (log-transformed, months), and cardiovascular disease

A dose–response relationship was evaluated by further subdividing Group 3 (FBG ≥ 130 mg/dL) into three categories (130–159 mg/dL, 160–189 mg/dL, and ≥ 190 mg/dL). Compared with the reference group (Group 2, 80–130 mg/dL, well controlled group), mortality risk increased progressively across these higher FBG ranges. The ≥ 190 mg/dL category demonstrated a significantly elevated risk, with an adjusted aOR) of 2.473 (95% CI: 1.544–3.981) (Table 3; Fig. 1).

**Fig. 2:**
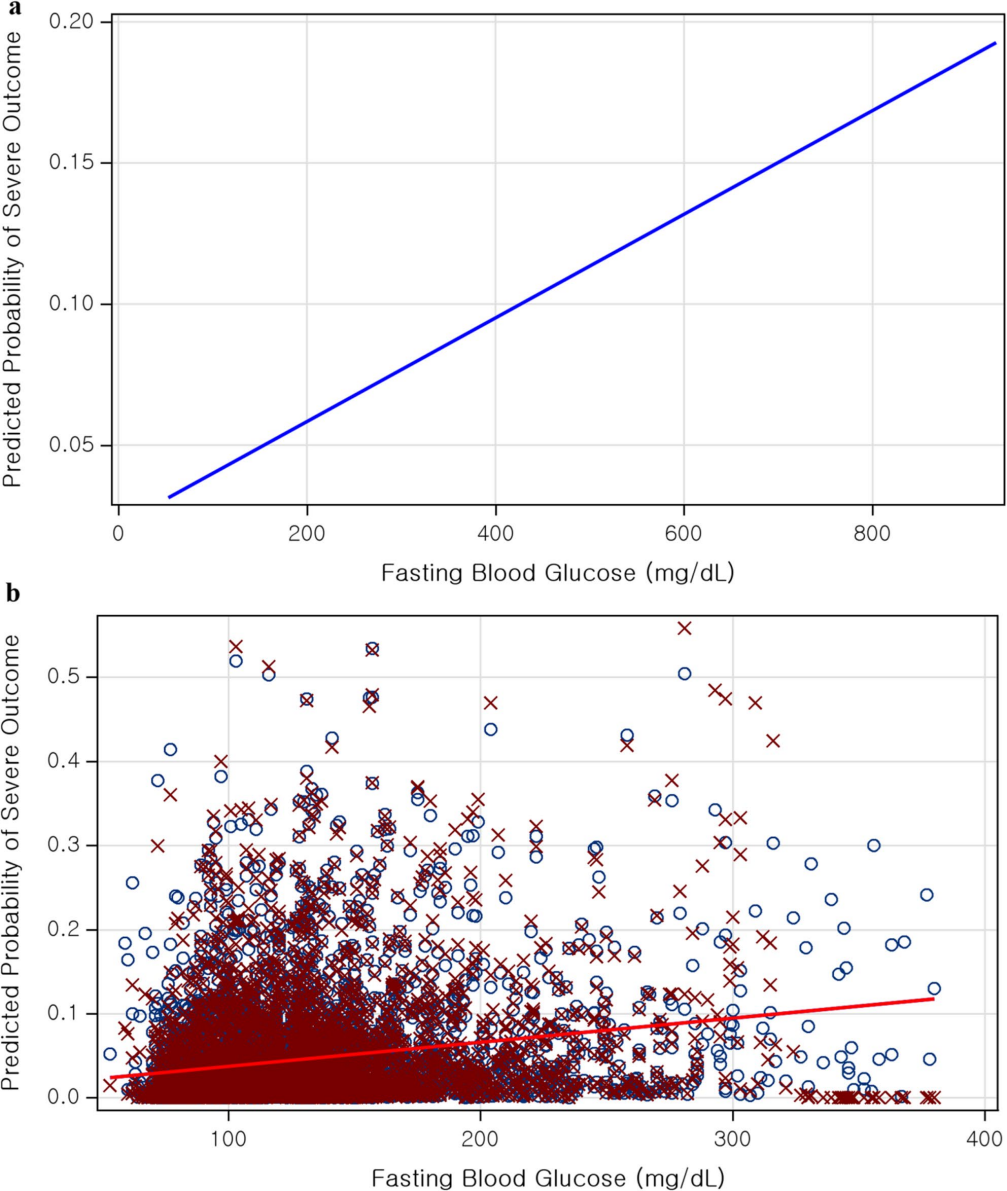
Dose–response relationship between FBG level and LRTI mortality. (**a**) Linear dose–response relationship estimated using a multivariable logistic regression model. Predicted mortality increased steadily as FBG rose. (**b**) Comparison of linear and restricted cubic spline models. Both models demonstrated similar increasing trends in predicted mortality across the FBG spectrum, with no evidence of a non-linear inflection point

A similar linear trend was confirmed in the linear regression model. For each 1 mg/dL increase in FBG, the risk of mortality increased by approximately 0.7% (aOR = 1.007, 95% CI: 1.004–1.010; β = 0.00680; *p* < 0.0001) (Fig. 2a).

RCS analysis also showed statistically significant results (*p* < 0.0001), with no evidence of a non-linear inflection point, indicating a predominantly linear relationship (Fig. 2b). In addition, RCS models using 3, 4, or 5 knots produced nearly identical curve shapes, further supporting the robustness and linearity of this association (Supplementary Figure S2).

Taken together, these results indicate that the association between FBG level and LRTI mortality follows a predominantly linear pattern, suggesting that increasing FBG is proportionally associated with higher mortality risk. A clear dose–response relationship was observed between FBG and mortality risk.

**Table 3:** Dose–response association between FBG categories and LRTI mortality

Fasting blood glucose category (mg/dL)	Number of participants (*n*)	Number of death (*n*, %)	Adjusted	
			ORs	(95% CI)
80–130	2464	97(3.94)	1.00	Ref.
130–159	972	51(5.25)	1.081	(0.723–1.615)
160–189	360	25(6.94)	1.448	(0.914–2.622)
≥ 190	414	30(7.25)	2.473	(1.544–3.961)

Note. Multivariable logistic regression analysis was performed after adjusting for age, sex, smoking status, physical activity, BMI, hypertension, household income decile, duration of diabetes (log-transformed, months), and cardiovascular disease. FBS categories were defined as follows: 80–130 mg/dL (reference), 130–159 mg/dL, 160–189 mg/dL, and ≥ 190 mg/dL

## Discussion

This study examined the association between FBG- used as an indicator of glycemic control- and mortality following LRTIs, including pneumonia and influenza, among adults with diabetes who developed LRTIs, using a nationally representative cohort from the Korean NHIS.

Among 4,514 diabetic patients with LRTIs, 212 deaths occurred (4.70%). Because the mortality rate was below 5%, the ORs are unlikely to substantially overestimate the true relative risk, allowing the aORs to be reasonably interpretated as approximations of relative risk.

Overall, patients whose FBG levels exceeded the recommended glycemic target (≥ 130 mg/dL) had a significantly higher risk of death, indicating that inadequate glycemic control is closely linked to worse infection outcomes.

The robustness of this association was confirmed through multiple analytic approaches. Outlier removal using the interquartile range (IQR) method yielded results consistent with the main analysis. Dose–response evaluation within the poorly controlled FBG group (≥ 130 mg/dL) showed a progressive increase in mortality from 130 to 159 mg/dL to 160–189 mg/dL and to ≥ 190 mg/dL. Analysis of FBG as a continuous variable further demonstrated a linear increase in mortality risk (aOR per 1 mg/dL increase = 1.007), supporting a graded, quantitative relationship. Restricted cubic spline analyses also showed a statistically significant trend, with no visually apparent non-linear inflection, and spline models using 3, 4, and 5 knots produced nearly identical curves. Collectively, these results support a consistent linear relationship between FBG levels and the risk of death from LRTIs.

These findings are biologically plausible and consistent with previous research. Hyperglycemia is known to impair innate and adaptive immune responses, including neutrophil chemotaxis and phagocytosis, macrophage activity, complement activation, cytokine signaling, and CD4 + T-cell and NK-cell function [2, 4]. Prior studies have reported higher 30-day and 90-day mortality from pneumonia in patients with type 2 diabetes [10], and older adults with diabetes experience higher rates of hospitalization, intensive care admission, and mortality from influenza [9]. During the 2009 H1N1 pandemic, diabetes increased ICU admission risk more than fourfold [7]. Evidence from COVID-19 further demonstrates that both diabetes and acute hyperglycemia predict severe outcomes and mortality [8, 28].

In a large Canadian cohort study, individuals with diabetes had up to a 2.17-fold higher risk of infection-related hospitalization and death compared with those without diabetes, with particularly pronounced differences for bacterial infections such as pneumonia and urinary tract infections [29]. In addition, studies in critically ill patients have shown that when blood glucose is tightly controlled within a range of 80–110 mg/dL, ICU and in-hospital mortality, as well as complications such as sepsis, renal failure, and multi-organ dysfunction, are significantly reduced [30]. Hyperglycemia itself, irrespective of diabetes status, has been associated with increased risks of mortality and heart failure [31]. Among patients with H1N1 pneumonia, higher fasting plasma glucose (FPG) levels were linked to lower oxygen saturation and greater disease severity, confirming FPG as an independent predictor of poor outcome [32], and in COVID-19, elevated blood glucose has likewise been identified as an important prognostic factor for severe clinical course and mortality [28]. Furthermore, although conducted in non-diabetic patients, prior work has shown that both very low (4.74 mmol/L) and very high (7.05 mmol/L) fasting glucose levels are associated with an increased risk of severe COVID-19 [33], indirectly supporting that patients who maintain glucose within the recommended target range may experience lower severity than those with uncontrolled hyperglycemia.

Consistent with prior evidence, the present study demonstrated that patients whose fasting glucose levels exceeded the recommended glycemic target had a significantly higher risk of mortality, and this risk increased in a linear pattern as FBG levels rose. This finding indicates a clear and quantitative relationship between glycemic control and mortality from LRTI. The additional calculation of absolute mortality proportions supplemented the relative measures, showing that increases in fasting glucose translated into clinically meaningful differences in mortality risk. Taken together, these consistent trends underscore the importance of adequate glycemic control in improving infection-related outcomes among individuals with diabetes.

Although some previous studies have reported increased risk at both low and high glucose levels [33], the present study did not observe a statistically significant elevation in risk among participants with fasting glucose below the target range. A population-based study from Taiwan reported a U-shaped association between fasting plasma glucose and LRTI-related mortality, with increased risks observed at both low (< 90 mg/dL) and high (> 200 mg/dL) glucose levels; however, the excess risk in the low-glucose range was attenuated after adjustment for comorbidities [34]. Across most Asian and Western cohort studies, poor glycemic control among individuals with diabetes has nonetheless been consistently associated with higher infection risk or mortality. This shared direction of association is biologically plausible, as hyperglycemia impairs innate and cell-mediated immune function and promotes pathogen proliferation. Accordingly, even in the presence of non-differential exposure misclassification, the direction of the association is likely to be preserved, with attenuation of effect size rather than reversal. Differences in secondary findings, including the presence or absence of a U-shaped relationship, may be explained by design-driven heterogeneity, differences in confounding control, and treatment context. Prior studies assessed baseline glucose levels before infection and included both individuals with and without diabetes, whereas the present study focused on post-infection mortality among patients with established diabetes. In addition, glucose-lowering medications may differentially influence infection outcomes. For example, SGLT2 inhibitor use has been associated with an increased reporting risk of ketoacidosis, which may exacerbate infection severity [35], whereas metformin has been shown to reduce pneumonia severity and mortality through anti-inflammatory and immunomodulatory effects [36]. These differences likely contribute to both the consistent overall direction and the observed heterogeneity across studies. In our analysis, the adjusted odds ratio for Group 1 (below-target FBG) was 0.630 (95% CI: 0.290–1.370), indicating a non-significant tendency toward lower severity. However, this result should be interpreted cautiously, as fasting glucose measurements were not obtained at the time of infection, and the small sample size in Group 1 limits statistical power. Future studies with larger samples or time-proximal glucose measurements may yield different or more conclusive findings.

### Several limitations should be acknowledged

First, FBG levels were obtained from the nearest health examination rather than at the time of infection. As a result, acute metabolic abnormalities such as stress-induced hyperglycemia could not be captured. Previous studies have shown that acute hyperglycemia increases mortality and cardiac complications regardless of diabetes status [31], but such acute effects could not be evaluated in this study.

Second, HbA1c and glycemic variability—key indicators of long-term glycemic control—were not available in the NHIS cohort. Prior research has demonstrated that intensive glycemic control (80–110 mg/dL) can reduce mortality and complications among critically ill patients [11, 30]. However, the absence of these metrics limits the ability to fully assess long-term glucose control and its association with infection severity.

Third, due to structural limitations of the NHIS database, lag-day analyses (e.g., glucose values 30 or 90 days before infection) and time-varying exposure analyses could not be performed. Health screening data are provided only in “year-month” format, preventing precise time alignment between infection onset and glucose measurements, and precluding continuous tracking of FBG. Consequently, reverse causation could not be fully excluded.

Fourth, the potential for selection and information bias remains. Health examination data are more likely to include relatively healthier individuals or those with higher screening participation, while patients with severe or unstable conditions may be underrepresented. As a result, severe cases may have been partially underestimated within the cohort.

Fifth, information on diabetes treatment (e.g., insulin or oral hypoglycemic agents), use of corticosteroids or other immunosuppressive medications, and vaccination status (influenza or pneumococcal) was not available in the NHIS health screening dataset and therefore could not be incorporated into the analysis. In addition, although major diabetes-related and cardiopulmonary comorbidities such as CVD, CKD, and COPD were adjusted for, more granular measures of infection severity (e.g., oxygen requirement or laboratory markers) could not be considered. Consequently, residual confounding related to treatment patterns, vaccination, and clinical severity may remain, and the observed associations should be interpreted with caution. Moreover, individuals with major comorbid conditions such as cancer and advanced chronic diseases were excluded due to small case numbers and concerns about statistical instability. Furthermore, when multiple infection episodes occurred, only the most severe episode was retained for analysis. These design choices may limit the generalizability of the findings, particularly to older or multimorbid populations, and may introduce selection effects toward more severe infection episodes.

Lastly, because this study was restricted to patients with diabetes who had already developed lower respiratory tract infections, the findings primarily inform post-infection prognosis and have limited external validity with respect to infection risk in non-diabetic or general populations.

### Despite these limitations, this study offers several important strengths

A major strength of this study is the use of the NHIS-NSC, a nationally representative dataset derived from Korea’s single-payer health insurance system. Because the Korean NHI provides universal coverage with standardized coding and unified claims processes, selection bias and misclassification are minimized, and patients can be followed longitudinally without loss to follow-up. The integration of medical utilization records, health screening data, mortality records, and socioeconomic information further ensures comprehensive exposure and outcome ascertainment. These features collectively enhance the validity and generalizability of our findings. In addition, because the NHIS health screening program is broadly implemented, the association between fasting glucose and mortality could be evaluated even among relatively healthy community-dwelling adults.

Second, this study demonstrated a clear and clinically meaningful association between elevated FBG (poorly controlled) and increased mortality risk from LRTIs in patients with diabetes. Using nationally representative real-world data, we not only confirmed the importance of adequate glycemic control but also identified a range in which mortality risk rises sharply. Specifically, the adjusted odds ratio increased to 1.4 at FBG levels of 160–189 mg/dL and to approximately 2.5 at ≥ 190 mg/dL compared with the preceding category. These findings are grounded in actual clinical practice patterns and are directly applicable to routine diabetes care in Korea.

Third, we observed a clear and consistent dose–response relationship between FBG and LRTI mortality risk. The dose–response analysis across FBG strata and multiple sensitivity analyses—including outlier removal and alternative adjustment models incorporating chronic comorbidities—demonstrated a highly consistent pattern. This robustness strengthens the credibility of the findings. The addition of absolute mortality proportions further complements the adjusted odds ratios, providing clinically meaningful interpretation. The results collectively suggest that glycemic control plays a key role in improving infection outcomes among patients with diabetes.

Future research should incorporate long-term glycemic indicators such as HbA1c, measures of glycemic variability, and acute metabolic parameters obtained at or near the time of infection. Additionally, examining interactions with key metabolic factors—such as body mass index (BMI), which has been emphasized as an important prognostic factor [28], and blood pressure—would help clarify complex pathways underlying infection severity. Such multifactorial approaches will support more comprehensive research on chronic metabolic conditions and their influence on infectious disease outcomes.

## Conclusion

This study demonstrates that poor glycemic control is a major and preventable contributor to LRTI related mortality in individuals with diabetes. The robust dose–response pattern—identified using real-world national data—indicates that even moderate elevations in fasting glucose confer substantial risk.

These results position glycemic control as an immediate and actionable priority in both primary care and infection management, with the potential to meaningfully reduce mortality among diabetic populations. Glycemic control should therefore be viewed not only as a chronic disease target but also as a critical component of infection prevention and management in routine care.

## Electronic Supplementary Material

Below is the link to the electronic supplementary material.

Supplementary Material 1

## Data Availability

Availability of data and materialsThe datasets used in this study are not publicly available due to strict data protection regulations of the National Health Insurance Service (NHIS). The raw data cannot be shared by the authors and are accessible only through the NHIS data analysis platform upon authorized request and approval by the NHIS.

## Abbreviations

ANOVA: Analysis of variance
AUC: Area under the curve
BMI: Body mass index
CI: Confidence interval
CKD: Chronic kidney disease
COPD: Chronic obstructive pulmonary disease
COVID-19: Coronavirus disease 2019
CVD: Cardiovascular disease
DM: Diabetes mellitus
FBS: Fasting blood sugar
FPG: Fasting plasma glucose
ICD: International classification of diseases
ICU: Intensive care unit
KCD: Korean Standard Classification of Diseases
LRTIs: Lower respiratory tract infections
NHI: National Health Insurance
NHIS: National Health Insurance Service
NHIS-NSC: National Health Insurance Service- National Sample Cohort
OR: Odds ratio
aOR: Adjusted odds ratio
RCS: Restricted cubic spline
ROC: Receiver operating characteristic
RR: Relative risk
VIF: Variance inflation factor
