# Supplementary material for: Glucose control and mortality from lower respiratory tract infections in patients with diabetes: evidence from real-world data
Source: BMC Infect Dis. 2026 Mar 14;26:806. doi: 10.1186/s12879-026-13045-8 (PMC13101151; doi:10.1186/s12879-026-13045-8)

Supplementary Table S1. Definitions of Exposure, Outcome, and Diabetes Status

| **Category** | **Variable / Definition** | **Details** |
| --- | --- | --- |
| Exposure | Fasting blood glucose control group at the time of infection | Group 1: <80 mg/dL Group 2: 80–129 mg/dL Group 3: ≥130 mg/dL |
|  | Measurement timing | The fasting glucose measurement closest to (and prior to) the index infection was used. |
| Outcome | Severity (death) | In-hospital death where the primary cause of death was respiratory infection (ICD-10 J09–J11 or J12–J18), or cases in which the primary diagnosis at the final claim was lower respiratory infection and the patient was recorded as deceased. |
| Population | Diabetes mellitus | Defined as any of the following:  (1) Self-reported diabetes history or current anti-diabetic medication use in health screening records  (2) FPG ≥ 126 mg/dL at any health examination. |

**Note.** This table provides standardized definitions of exposure, outcome, and diabetes status used in the analysis. All definitions were derived from NHIS claims and health screening records to ensure reproducibility and consistency across analyses.

Supplementary Table S2. Definitions of Confounders and Comorbidities

| **Category** | **Variable / Definition** | **Details** |
| --- | --- | --- |
| Demographics | Age | Adults aged ≥ 20 years. |
|  | Sex | Male / Female (from NHIS eligibility data). |
|  | Income level | Deciles 1–10 from NHIS health screening socioeconomic variables. |
| Health  behaviors | Smoking status | Never / Former / Current smoker (self-reported in health screening). |
| Anthropometrics | BMI category | Underweight, Normal, Pre-obesity, Obesity (as defined in Methods) (18) |
| Blood pressure | Hypertension | Yes/No based on health screening questionnaire and blood pressure criteria (as defined in Methods). |
| Other health conditions | Previous pulmonary tuberculosis | Self-reported “currently under diagnosis or treatment for TB” in health screening records. |
|  | Cancer or other chronic disease treatment | Self-reported “yes” to ongoing cancer or other chronic disease treatment (NHIS questionnaire; specific disease types not disclosed). |
| Comorbidities (claims-based) | Chronic kidney disease (CKD) | ICD-10 N18 recorded as primary diagnosis between diabetes diagnosis and index infection. |
|  | Cardiovascular disease (CVD) | ICD-10 I20–I25, I50, I63–I69 as primary diagnosis after diabetes diagnosis and before infection. |
|  | Chronic obstructive pulmonary disease (COPD) | ICD-10 J43–J44 within 3 years prior to the infection date. |

**Note.** This table summarizes the operational definitions of confounders and comorbidities used in the analysis. Definitions were derived from NHIS health screening questionnaires and claims data to ensure consistency and reproducibility across models.

Supplementary Table S3. Baseline characteristics (data were not shown in manuscript)

| Variables | N (%) | Group 1  N (%) | Group 2  N (%) | Group 3  N (%) | p-value | |
| --- | --- | --- | --- | --- | --- | --- |
| Household  income decile |  |  |  |  |  | |
| 1st decile | 453(10.21) | 36(12.63) | 245(9.71) | 173(10.14) | 0.1404 | |
| 2nd -3rd decile | 624(14.03) | 40(14.04) | 342(13.56) | 242(14.19) |  |  |
| 4th -5th decile | 772(17.36) | 37(12.98) | 415(16.45) | 320(18.76) |  |  |
| 6th -7th decile | 886(19.92) | 48(16.84) | 524(20.77) | 314(18.41) |  |  |
| 8th decile | 491(11.04) | 39(13.68) | 283(11.22) | 169(9.91) |  |  |
| 9th decile | 607(13.65) | 42(14.74) | 332(13.16) | 233(13.66) |  |  |
| 10th decile | 614(13.80) | 41(14.39) | 344(13.63) | 229(13.42) |  |  |
| Pulmonary tuberculosis  medication history |  |  |  |  |  |  |
| Yes | 5(0.11) | 0(0.00) | 5(0.20) | 0(0.00) | 0.1388 |  |
| No | 4,509(99.89) | 285(100.00) | 2,518(99.80) | 1,706(100.00) |  |  |
| Cancer or other disease |  |  |  |  |  |  |
| Yes | 84(1.86) | 10(3.51) | 56(2.22) | 18(1.06) | 0.0024^*^ |  |
| No | 4,430(98.14) | 275(96.49) | 2,467(97.78) | 1,688(98.94) |  |  |
| Chronic kidney disease |  |  |  |  | 0.0006^*^ |  |
| Yes | 108(2.39) | 5(1.75) | 43(1.70) | 60(3.52) |  |  |
| No | 4,406(97.61) | 280(98.25) | 2,480(98.30) | 1,646(96.48) |  |  |
| Chronic obstructive pulmonary disease |  |  |  |  |  |  |
| Yes | 272(6.03) | 14(4.91) | 152(6.02) | 106(6.21) | 0.6942 |  |
| No | 4242(93.97) | 271(95.09) | 2,371(93.98) | 1,600(93.79) |  |  |
| Total | 4514  (100.00) | 285  (6.31) | 2523  (55.89) | 1706  (37.79) |  |  |

**Note.** Chi-square test. Values are presented as number (%). Group classification was based on FBS levels: Group 1 (< 80 mg/dL), Group 2 (80–130 mg/dL), and Group 3 (≥ 130 mg/dL).

Supplementary Table S4. Predicted absolute mortality risk and absolute risk differences across fasting plasma glucose categories

| Group | Predicted Mortality Risk  (%) | Absolute Risk Difference vs Reference (%) |
| --- | --- | --- |
| Group 1 | 2.24 | -1.61 |
| Group 2 | 3.85 | reference |
| Group 3 | 6.00 | + 2.15 |

**Note.** Predicted absolute mortality risks and risk differences were estimated using the fully adjusted logistic regression model and rounded to two decimal places. Absolute risk differences were calculated relative to the reference group (80–130 mg/dL).

Supplementary Table S5. Sensitivity analysis after outlier exclusion

| Group | Number of participants (n) | Number of death (n, %) | Crude | | Adjusted | |
| --- | --- | --- | --- | --- | --- | --- |
|  |  |  | ORs | (95% CI) | ORs | (95% CI) |
| Group 1 | 285 | 7(2.46) | 0.630 | (0.290–1.370) | 0.536 | (0.225–1.276) |
| Group 2 | 2523 | 97(3.84) | 1.00 |  | 1.00 |  |
| Group 3 | 1678 | 106(6.32) | 1.686 | (1.271–2.237) | 1.542 | (1.111–2.140) |

**Note.** Multivariable logistic regression analysis was performed after adjusting for age, sex, smoking status, physical activity, BMI, hypertension, household income decile, duration of diabetes (log-transformed, months), and cardiovascular disease. Group classification was based on FBS levels: Group 1 (< 80 mg/dL), Group 2 (80–130 mg/dL), and Group 3 (≥ 130 mg/dL). Outliers above 383 mg/dL (Q3 + 1.5 × IQR) were excluded to verify the robustness of the results.

Supplementary Table S6. Sensitivity analysis with additional comorbidity adjustment

| Group | Number of participants (n) | Number of death (n, %) | Crude | | Adjusted | |
| --- | --- | --- | --- | --- | --- | --- |
|  |  |  | ORs | (95% CI) | ORs | (95% CI) |
| Group 1 | 285 | 7(2.46) | 0.630 | (0.290–1.370) | 0.576 | (0.242–1.370) |
| Group 2 | 2523 | 97(3.84) | 1.00 |  | 1.00 |  |
| Group 3 | 1706 | 108(6.33) | 1.690 | (1.276–2.240) | 1.551 | (1.118–2.151) |

**Note.** Multivariable logistic regression analysis was performed after adjusting for age, sex, smoking status, physical activity, BMI, hypertension, household income decile, duration of diabetes (log-transformed, months), cardiovascular disease, cancer, pulmonary tuberculosis, CKD, and COPD.

Group classification was based on FBS levels: Group 1 (< 80 mg/dL), Group 2 (80–130 mg/dL), and Group 3 (≥ 130 mg/dL).

Supplementary Table S7. Combined sensitivity analyses with outlier exclusion and extended adjustment

| Group | Number of participants (n) | Number of death (n, %) | Crude | | Adjusted | |
| --- | --- | --- | --- | --- | --- | --- |
|  |  |  | ORs | (95% CI) | ORs | (95% CI) |
| Group 1 | 285 | 7(2.46) | 0.630 | (0.290–1.370) | 0.576 | (0.242–1.370) |
| Group 2 | 2523 | 97(3.84) | 1.00 |  | 1.00 |  |
| Group 3 | 1678 | 106(6.32) | 1.686 | (1.271–2.237) | 1.556 | (1.120–2.163) |

**Note.** Multivariable logistic regression analysis was conducted after adjusting for age, sex, smoking status, physical activity, BMI, hypertension, household income decile, duration of diabetes (log-transformed, months), and cardiovascular disease. Group classification was based on fasting blood glucose (FBS) levels: Group 1 (< 80 mg/dL), Group 2 (80–130 mg/dL), and Group 3 (≥ 130 mg/dL). Outlier exclusion: Values above 383 mg/dL (Q3 + 1.5 × IQR) were removed to assess robustness. Extended adjustment: Additional confounders included cancer (1.86%, n = 84), pulmonary tuberculosis (0.11%, n = 5), chronic kidney disease (N18) and renal failure (N19) (2.39%, n = 108), and chronic obstructive pulmonary disease within the past 3 years (J43–J44) (6.03%, n = 272).

Supplementary Figure S1. Receiver operating characteristic (ROC) curve for the fully adjusted logistic regression model

*(insert Supplementary Figure S1 here)*

Note. Receiver operating characteristic (ROC) curve illustrating the discrimination performance of the fully adjusted logistic regression model for predicting in-hospital mortality. The area under the curve (AUC) was 0.8218, demonstrating good discriminatory ability.

Supplementary Figure S2. Comparison of restricted cubic spline models using 3, 4, and 5 knots

*(insert Supplementary Figure S2 here)*

Note. Predicted probabilities of severe outcomes (in-hospital death) were estimated using logistic regression models with fasting blood glucose as a continuous variable. Restricted cubic spline (RCS) models were fitted using 3 knots (10th, 50th, 90th percentiles), 4 knots (5th, 35th, 65th, 95th percentiles), and 5 knots (5th, 27.5th, 50th, 72.5th, 95th percentiles). The solid lines represent model-based predicted probabilities, and the points represent observed values. All models showed similar linear trends without evidence of non-linearity.


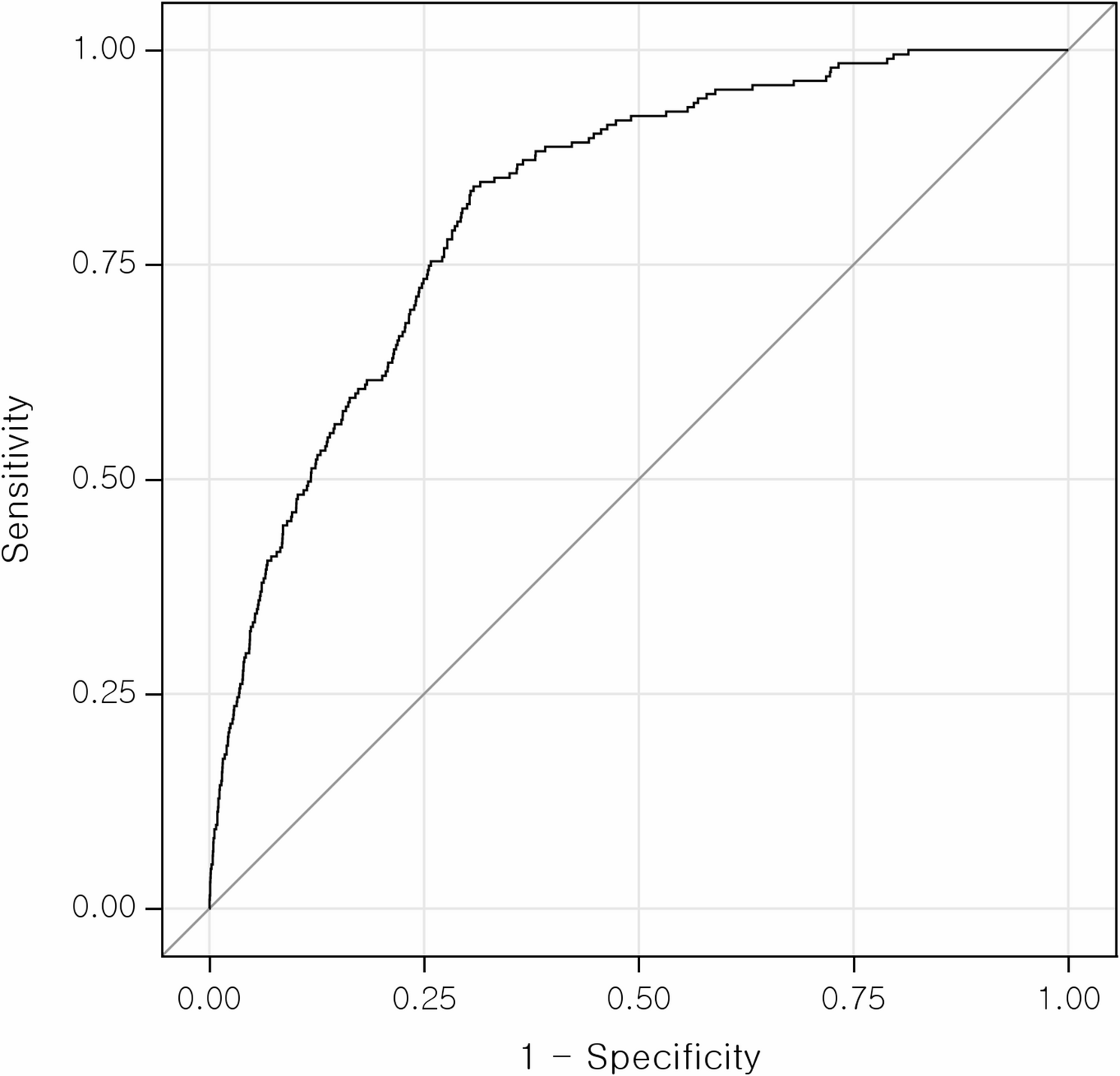


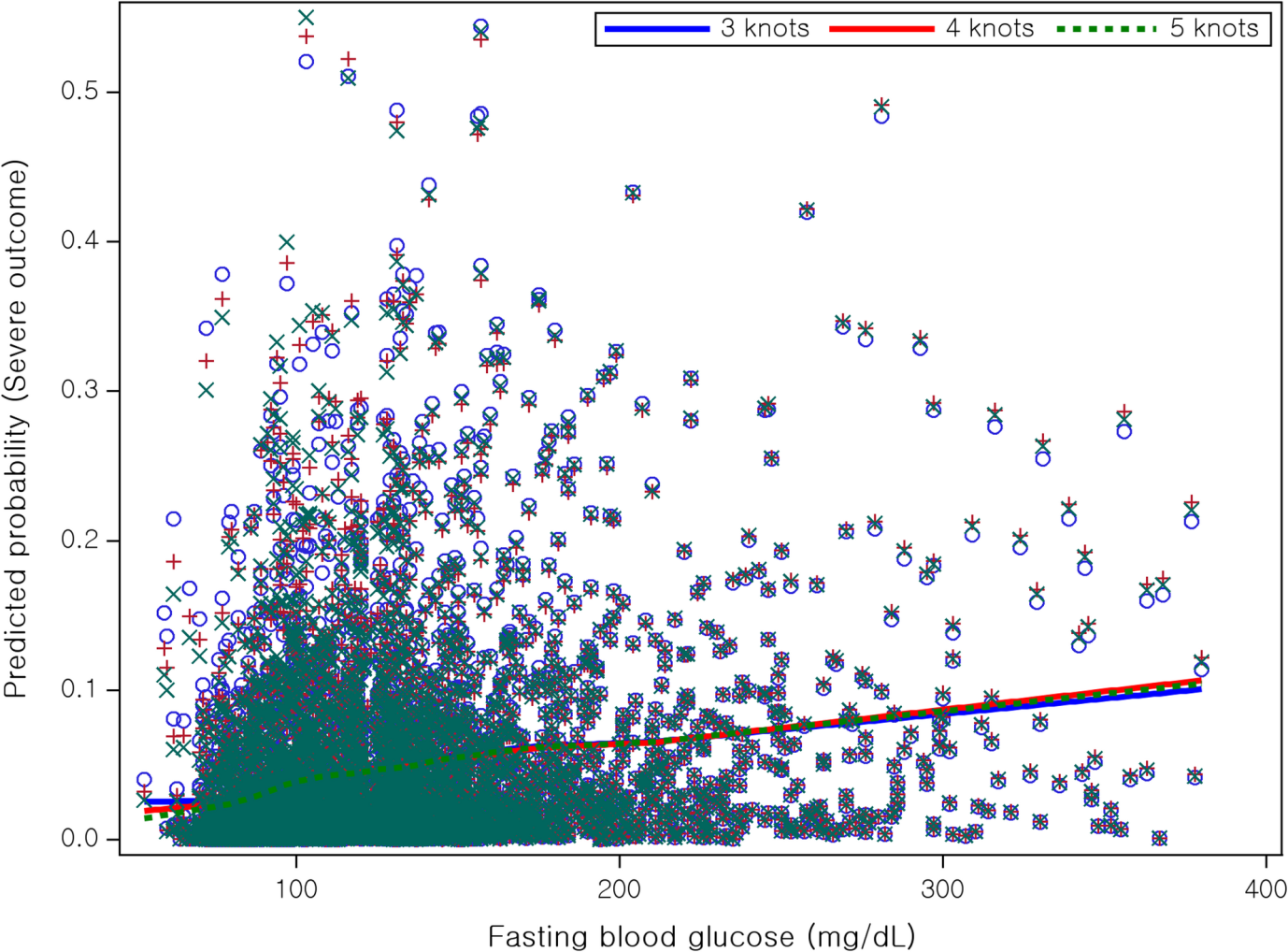

Supplement: Supplementary file 1 — Supplementary Material 1 [file 12879_2026_13045_MOESM1_ESM.docx]
